# Utilization of Bioelectrical Impedance to Predict Intramuscular Fat and Physicochemical Traits of the Beef *Longissimus Thoracis et Lumborum* Muscle

**DOI:** 10.3390/foods9060836

**Published:** 2020-06-25

**Authors:** João Afonso, Cristina Guedes, Virgínia Santos, Raul Morais, José Silva, Alfredo Teixeira, Severiano Silva

**Affiliations:** 1 Faculdade de Medicina Veterinária, ULisboa, Avenida da Universidade Técnica, 1300-477 Lisboa, Portugal; 2 Centro de Ciência Animal e Veterinária, Universidade de Trás-os-Montes e Alto Douro, 5001-801 Vila Real, Portugal; cguedes@utad.pt (C.G.); vsantos@utad.pt (V.S.); jasilva@utad.pt (J.S.); ssilva@utad.pt (S.S.); 3 INESC TEC-INESC Technology and Science and Universidade de Trás-os-Montes e Alto Douro, 5001-801 Vila Real, Portugal; rmorais@utad.pt; 4 CIMO, Instituto Politécnico de Bragança, 5300-253 Bragança, Portugal; teixeira@ipb.pt

**Keywords:** bioelectrical impedance analysis, meat quality, beef

## Abstract

The bioelectrical impedance analysis (BIA) is a non-destructive technique that has been successfully used to assess the body and carcass composition of farm species. This study aimed to predict intramuscular fat (IMF) and physicochemical traits in the longissimus thoracis et lumborum muscle (LM) of beef, using BIA. These traits were evaluated in LM samples of 52 crossbred heifer carcasses. The BIA was performed in LM, using a 50 Hz frequency high precision impedance converter system. A correlation analysis of the studied variables was performed. Then a stepwise with a k-folds cross validation procedure was used to modelling the prediction of IMF and physicochemical traits from BIA parameters (24.5% ≤ CV ≤ 47.3%). Wide variation was found for IMF and BIA parameters. In general, correlations of BIA parameters with IMF and physicochemical traits were moderate to high and were similar for all BIA parameters (−0.50 ≤ r ≤ 0.50 only for total pigments, a* and pH48). It was possible to predict IMF and physicochemical traits from BIA. The best fit explained 79.3% of the variation in IMF, while for physicochemical traits the best fits were for sarcomere length and shear force (64.4% and 60.5%, respectively). The results confirmed the potential of BIA for objective measurement of meat quality.

## 1. Introduction

Meat is a nutritious and popular food, which provides high biological value proteins and important micronutrients, and in recent years increasing attention has been put on its quality and safety [1]. Meat quality is affected by several factors throughout the production line and identifying and ensuring meat quality remains an ongoing challenge for meat processors [2]. The traditional evaluation of meat quality such as intramuscular fat (IMF) and physicochemical traits involves destructive, expensive and time-consuming methods [3]. To overcome these limitations, several non-destructive, precise, and fast techniques have been developed and successfully applied in meat quality assessment. Over the past two decades, there have been several emerging techniques, particularly those based on imaging and spectroscopic principles [4,5]. Among the imaging techniques, computed tomography [6], computer vision [7], and ultrasound [5] stand out. Concerning spectroscopic techniques, the emphasis has been put on near-infrared spectroscopy (NIRS) techniques [8,9]; hyperspectral imaging systems [10,11], and Raman spectroscopy [2]. For all these techniques, attributes of accuracy, precision, cost, and ease of use have been considered. However, some attributes, particularly those related to cost and ease of use, are not achieved in most of the previously mentioned techniques. This encourages further research to investigate the application of other techniques in the assessment of meat quality traits.

The bioelectrical impedance analysis (BIA) is an example of an unexpansive and easy to use technique that has been used successfully to assess the body and carcass composition of several farm species [12,13,14]. However, not much information has been reported on the ability of such technique to assess IMF and/or physicochemical meat quality traits. Therefore, our objective was to examine the usefulness of BIA to predict IMF and physicochemical traits of the beef Longissimus thoracis et lumborum muscle.

## 2. Materials and Methods

Fifty-two Limousine x Holstein crossbred heifers with 355.5±35.0 kg of live weight and with age ranging from 9–12 months were slaughtered at the PEC Nordeste (Penafiel, Portugal) abattoir. All animals were cared for and killed in compliance with the welfare regulations and respecting EU Council Regulation (EC) No. 1099/2009. The animals were stunned using captive-bolt pistol prior to exsanguination and dressed according to standard commercial practices. The transport time of the animals from the farm to the slaughterhouse was 1–2 h and the pre-slaughter resting duration in lairage was approximately 3 h.

### 2.1. Muscle Sample Preparation

After slaughter, the carcasses were dressed and centrally-split into two sides. Then entered the chill room and were refrigerated for two days at 2 °C. After the chill period, carcasses were deboned in accordance with the scheme used by the abattoir, whereas no primal cuts were removed prior to deboning. During this process, using a metallic ruler, a cut of 10 cm length of LM at the level of 3rd and 5th lumbar vertebrae was removed from the carcass. This cut was divided into two slices, which were identified, weighed, individually vacuum-packed, placed in insulated cooled containers and transported to the laboratory, for an aging period of ten days, at 2 °C. After this period the BIA and physicochemical analyses were carried out, and the corresponding results recorded.

### 2.2. Bioelectrical Impedance Analysis

After removal of subcutaneous and intermuscular fat, one 3 cm thick slice sample of LM from each carcass was placed over a flat surface for BIA measurements. The bioelectrical impedance device used in the present study was a single frequency unit built specifically for this purpose, around a high precision impedance converter AD5933 system (Analog Devices, Norwood, MA, USA). After calibration, and at a frequency of 50 kHz, the magnitude of the impedance and relative phase of the impedance was calculated, using a Discrete Fourier Transform algorithm to obtain the resistance (Rs) and reactance (Xc) values. The impedance (Z) was calculated as Z = (Rs^2^ + Xc^2^)^0.5^. As detector terminals, two hypodermic needles were inserted 2 cm into each 3 cm thick LM slice, 10 cm apart, providing an attachment for the connecting clips of the bioelectrical impedance terminal leads. For each sample, four measurements were made and the values of Rs and Xc correspond to their average. All bioelectrical impedance measurements were made in LM slices with temperature ranging between 2 and 6 °C.

### 2.3. Intramuscular Fat

For IMF chemical determinations, vacuum-packed samples were unpacked and intermuscular and the subcutaneous fats were carefully trimmed from the LM samples. The samples were minced and homogenized, using an Ultra Turrax T25 apparatus (IKA, Germany), prior to the chemical analysis. Chemical IMF content of LM samples was obtained in triplicates after ether-extraction in a Tecator Soxtec HT 1043 (Höganäs, Sweden), using petroleum ether as the solvent, and was determined gravimetrically, after evaporating the solvent according to AOAC (2000) [15] procedure. The content of the chemical IMF was expressed as a percentage of LM weight.

### 2.4. Total Collagen

Two sub-samples of approximately 4 g were obtained from each meat sample. The sub-samples were finely chopped, while frozen, and each one put into a flask with a lid. To each flask were added 30 mL of 7 N sulfuric acid. The flasks were closed and placed in an oven at 105 °C for 16 h, and the sample was hydrolyzed. Then, the hot hydrolyzate was transferred to 200 mL volumetric flasks, the flasks being washed with distilled water and filled to a volume of 200 mL. After homogenization, the contents of the flasks were filtered on Whatman filter paper No. 4, up to a volume of 100 mL, and stored at a temperature of approximately 4 °C, for some time that did not exceed two weeks. The previous filtrate was pipetted and diluted in distilled water, in 100 mL flasks, so that the concentration of L-hydroxyproline in the final dilution was between 0.6 and 2.4 µg/mL. To 2 mL of the final dilution, 1 mL of an oxidizing solution of chloramine T was added, homogenized and left to stand for 20 min at room temperature. Then, 1 mL of colorimetric reagent was added and homogenized, 25 mL of which was prepared by diluting 2.5 g of 4-dimethylaminobenzaldehyde in 8.75 mL of 60% perchloric acid to which it was added, slowly with stirring, 16.25 mL of 2-propanol. The covered test tubes were quickly placed in a Digiterm 100 water bath (J.P. Selecta, Barcelona, Spain) at 60 °C for exactly 15 min. Then they were quickly cooled under running water for at least 3 min. Absorbance was measured at 558 nm against a blank 1 cm cell optical path assay on a Jasco V-530 UV/VIS spectrophotometer (JASCO Corporation, Tokyo, Japan). The absorbance of 0.6, 1.2, 1.4, and 2.4 µg/mL L-hydroxyproline solutions were also measured in order to construct the standard curve for each assay. For the preparation of L-hydroxyproline solutions, an intermediate solution of L-hydroxyproline with 6 µg/mL was initially prepared, pipetting 5 mL of the L-hydroxyproline stock solution (600 µg/mL) into a 500 mL flask and making up the remaining volume with distilled water. From this solution, solutions with four different concentrations of L-hydroxyproline (0.6, 1.2, 1.4, and 2.4 µg/mL) were then prepared, having been pipetted 10, 20, 30, and 40 mL. To, respectively, of the intermediate solution, for 100 mL. To flasks, making up the remaining volume of the flasks with distilled water. After obtaining the standard curve, the concentration of L-hydroxyproline (µg/mL. To) of each sample was calculated using the corrected absorbance. The concentration of total collagen in the sub-samples was obtained by multiplying the concentration of L-hydroxyproline by factor 7.25 [16].

### 2.5. Total Pigments

Total pigments were determined using the method of Wierbicki et al. (1955) [17] modified by Boccard et al. (1981) [18]. In this method, total pigments are dosed as cyanmetamyoglobin. Approximately 5.0 g was obtained in duplicate of each meat sample of frozen chopped muscle, to which 40 mL of cold distilled water were added and homogenized at a rapid speed for 30 s with Ultra-Turrax T25B (Kika Labortechnik, Staufen, Germany). The pH of each sample was checked and samples with a pH higher than 6.0 were acidified with 0.1 M HCl to pH 5.5. The homogenate was filtered on medium porosity paper with a high retention time (Whatman No. 3), with the additional use of 5 mL of distilled water to remove all residues of the homogenate from the flask for filtration. Being the filtrate perfectly translucent and red, 25 mL of the filtrate was transferred to the test tube. Otherwise, the filtrate obtained was re-filtered and, in case the situation remained, the procedure was repeated. A test tube with an equal amount of distilled water was prepared for the blank test. Then, 0.1 mL of 2.5% potassium ferricyanide solution (K_3_Fe(CN)_6_) was added to each of the tubes and homogenized. Afterwards, 0.1 mL of 5% potassium cyanide (KCN) was added to each of the tubes and homogenized, and the solution should turn red due to the formation of cyanmethamyoglobin. The absorbance was measured at 540 nm, in a 1 cm optical cell, in a Jasco V-530 UV/VIS spectrophotometer (JASCO Corporation, Tokyo, Japan). The concentration of myoglobin in the fresh product (mg/g) was obtained by multiplying the corrected absorbance by 14.56; this factor is an empirical value for the conditions in which the analysis was carried out and for a given standard curve.

### 2.6. Cooking Losses

From each sample, 70–100 g of meat was weighed, and a Digi-Sense Thermocouple Scanning Thermometer (Cole Parmer, Niles, IL, USA) was placed in the thermal center of each meat block. Each meat block was then put in a plastic bag which was closed in order to remove the air, to facilitate immersion, preventing water from entering and moving the probe, and was placed in a Digiterm 100 water bath (JP Selecta, Barcelona, Spain) at 75 °C, until the internal temperature of 70 °C was reached. Subsequently, it was placed in running water until the internal temperature reached 15 °C. The meat was removed from the bag, dried carefully and weighed, thus obtaining the final weight. Cooking losses were determined by the difference between the initial weight (W_i_) and the final weight (W_f_) after cooking, and were expressed as a percentage of the initial weight [19]:

Cooking losses (%) = [(W_i_ − W_f_)/W_i_] × 100. (1)

These samples were subsequently packaged and stored at 4 °C to be used in determining the shear force.

### 2.7. Sarcomere Length

To determine the length of the sarcomeres was used the method described by Cross et al. (1981) [20]. Briefly, approximately 2 g of LM was cut with a scalpel in small portions, to which 30 mL of a cold 0.25 M sucrose solution was added and subsequently homogenized at slow speed for 60 seconds with the Ultra-Turrax T25B (Kika Labortechnik, Staufen, Germany). When the fibers were not sufficiently broken, they were further homogenized for 20 to 30 s. This procedure was repeated with the care that the obtained myofibrils had no less than 10 sarcomeres. A drop of the homogenate was placed on a slide, using a Pasteur pipette, and it was covered with a coverslip, having been observed under the Nikon Labophot-2 optical microscope in phase 1, with the 40× objective with attached camera. The length of 10 consecutive sarcomeres was measured, and approximately 15 groups of 10 sarcomeres per sample were measured using the image analysis software Matrox Inspector 4.1 (Matrox Electronic Systems Ltd., Dorval, QC, Canada). The average length of the sample sarcomeres was subsequently determined.

### 2.8. Warner–Bratzler Shear Force

Meat samples used to determine the cooking losses were removed from the refrigerator and placed at room temperature for temperature equilibrium. Approximately ten sub-samples of meat were cut in cuboid shape with 1 cm^2^ of side and about 3–5 cm in length. The cut was made so that the muscle fibers were parallel to the length of the cuboid. The sub-samples were placed in the Stevens QTS25 Texturometer tray with the Warner–Bratzler rectangular hole probe (Stevens Advanced Weighing Systems Ltd., Great Dunmow, UK), with the fibers arranged perpendicularly to the blade direction, and the probe was used at a speed of 100 mm/min [21]. The mean values of the maximum shear force (kg/cm^2^) were then obtained.

### 2.9. Color and pH Measurements

Color and pH measurements were made 48 h after slaughter. The LM surface color measurements were obtained with the handheld CR-10 colorimeter (Konica Minolta Sensing Inc., Osaka, Japan) and assessed using the L*, a*, and b* three-dimensional color space, defined by the Commission Internationale de l`Éclairage [22]. In this system, L*, a*, and b* represent the measurements of luminosity, red-green, and yellow-blue, respectively. The LM slices were held in open plastic bags at 2 °C for two hours (blooming time) to ensure optimal myoglobin oxygenation [23]. Then, the determinations of L* a* and b* parameters were carried out randomly, in three locations at the cutting surface of the LM slices, using measurement geometry 0°/45°, D65 illuminant and 10° observer. The colorimeter was calibrated before the usage with a standard white ceramic disk and a trap opening for black. The average pH values resulted from three readings performed directly on each LM slice, using a drilling electrode coupled to a Hanna HI 9025 pH meter (Hanna Instruments, Woonsocket, RI, USA) with automatic temperature compensation.

### 2.10. Statistical Analysis

A descriptive analysis of the data was performed, using Microsoft Excel, to obtain the mean, standard deviation, maximum value, minimum value and coefficient of variation of the variables under study. Correlations were established between the BIA parameters and IMF and the physicochemical characteristics of the LM. A stepwise regression with k-fold cross-validation procedure was used for modelling of the predictions of IMF and physicochemical characteristics of the LM with BIA parameters. The accuracy of the estimates was based on the k-fold coefficient of determination (k-fold-*R*^2^), while the root mean square error of the cross-validation (RMSE) was used to determine the precision of the prediction model. Additionally, as an indicator of the overall prediction ability of k-fold cross-validation models, the ratio of prediction to deviation (RPD) was evaluated. The RPD is calculated as the ratio of standard deviation (SD) of the reference values to the RMSE (RPD = SD/RMSE). An RPD > 2.5 indicates excellent prediction models, 2.0 < RPD < 2.5 indicates very good prediction models, 1.8 < RPD < 2.0 indicates good prediction models still allowing quantitative predictions, 1.4 < RPD < 1.8 indicates fair prediction models, still useful for assessment, and 1.0 < RPD < 1.4 indicates poor prediction models [24]. All statistical procedures were carried out using the John’s Macintosh Project (JMP) software version 14 (SAS Institute, Cary, NC, USA).

## 3. Results

As shown in Table 1, there was a low coefficient of variation (CV) for LW, dressing and HCW (respectively 9.8%, 2.2%, and 8.7%) but, with the exception of pH 48 h after slaughter (pH48; CV = 4.0%), all the other LM physicochemical traits and all BIA parameters showed higher CV (12.5% ≤ CV ≤ 47.3%). Most physicochemical traits showed a CV between 4.0% and 27.2%, lower or quite similar to the CV for the BIA parameters (24.5% ≤ CV ≤ 31.4%). The exception was the CV of 47.3% for IMF, with the IMF content of the samples ranging between 1.65% and 16.53%.

As shown in Table 2, with the exception of the moderate correlations of WBSF with total collagen (r = 0.697; *p <* 0.01) and sarcomere length (r = −0.569; *p* < 0.01), the other correlations between IMF, total collagen, sarcomere length and WBSF were all high – positive between IMF and sarcomere length (r = 0.830; *p* < 0.01) and negative in the remaining cases (−0.869 ≤ r ≤ −0.740; *p* < 0.01). All the other correlations between the different LM traits, although significant, were negligible to moderate (−0.707 ≤ r ≤ 0.708; *p* < 0.01).

Overall, the correlations with LM physicochemical traits were quite similar for the three BIA parameters (Table 3). Even so, Rs was the BIA parameter showing the highest correlation with IMF (r = 0.854; *p* < 0.01), WBSF (r = −0.778; *p* < 0.01), b* (r = 0.610; *p* < 0.01) and total pigments (r = 0.388; *p* < 0.01), Xc was the BIA parameter showing the highest correlation with sarcomere length (r = 0.803; *p* < 0.01), L* (r = 0.567; *p* < 0.01) and pH48 (r = −0.489; *p* < 0.01), and Z was the BIA parameter showing the highest correlation with total collagen (r = −0.716; *p* < 0.01), cooking losses (r = −0.607; *p* < 0.01) and a* (r = 0.431; *p* < 0.01). All BIA parameters were significantly correlated with LM physicochemical traits, although the correlations of BIA parameters with cooking losses and pH48 (−0.607 ≤ r ≤ −0.460; *p* < 0.01), and with total pigments, L*, a*, and b* (0.361 ≤ r ≤ 0.610; *p* < 0.01) were poor to moderate. The correlations with BIA parameters were high and negative for total collagen and WBSF (−0.778 ≤ r ≤ −0.705; *p* < 0.01), and high and positive for IMF and sarcomere length (0.748 ≤ r ≤ 0.854; *p* < 0.01).

The best multiple regression models for predicting physicochemical traits of LM, obtained by the stepwise regression procedure, are presented in Table 4. The multiple regression analysis of the data showed that a model with Rs and Xc explained 79.3% of the variation observed in IMF, with an RMSE of 1.631 and an RPD of 2.2. The main predictor was Rs, but the inclusion of Xc in the model increased the accuracy of the prediction by about six percentage points. The models for estimation of the other physicochemical traits showed much lower accuracy. Nevertheless, while the models for estimation of cooking losses, total pigments, L*, a*, and b*, and pH48 only explained 13.4–36.0% (*p* < 0.01) of the variation observed in these traits, the models for estimation of total collagen, sarcomere length and WBSF still explained a considerable amount of the variation observed, respectively 51.3%, 64.4%, and 60.5% (*p* < 0.01).

## 4. Discussion

The value now obtained for the correlation between sarcomere length and WBSF is in line with the correlations obtained by Muchenje et al. (2008) [25] for the same traits (r = −0.47 and −0.58, respectively for meat aged for 2 and 21 days; *p* < 0.05). However, with this exception, the results obtained by Muchenje et al. (2008) [25] are quite different from the present ones, with those authors reporting that most meat quality traits were not correlated (*p* > 0.05), while the present study showed significant correlations between all meat quality traits analyzed. The negative correlation now obtained between IMF and total collagen is in agreement with Ponnampalam et al. (2015) [26].

Unlike the present study, Silva et al. (1999) [27] reported the absence of a significant correlation between WBSF and IMF, but this may be related to the low level of IMF (1.9%) in the samples tested by those authors. Silva et al. (1999) [27] also reported the absence of a significant correlation between WBSF and total collagen, as well as Maher et al. (2005) [28], but the positive correlation now shown between these two traits is close to the one observed by Torrescano et al. (2003) [29]. Torrescano et al. (2003) [29] showed no significant correlation between sarcomere length and WBSF, but other authors have already shown a negative correlation between sarcomere length and WBSF (e.g., Ahnström et al., 2006 [30], and Silva et al., 1999 [27]) and, in fact, muscles with short sarcomere length are generally tough [31].

The significant correlations now obtained, between BIA parameters and all the LM physicochemical traits analyzed, confirmed the relationship, already shown by previous studies, between impedance and meat quality traits. In the case of IMF, total collagen, WBSF and sarcomere length, the high correlations now verified between these traits were reflected in moderate to high correlations of BIA parameters with the same four traits. Moreover, the sign of these correlations with BIA parameters reflected the sign of the correlations between the different meat quality traits correctly, except in the case of a*, that just showed a negligible correlation with BIA parameters.

Being fat an electrical insulator, the fat content of meat samples influences their bioelectrical impedance and Slanger and Marchello (1994) [32] developed a prediction equation that explained 83% of the variation observed in percentage fat of LM. Madsen et al. (1997) [33], using their patented apparatus for measuring the IMF in carcasses or parts of carcasses, or the total content of fat in minced meat, explained 70.6% and 86.5% of the variation observed in IMF, respectively with one and two insertions. Damez and Clerjon (2013) [34] refer the lack of consistency in the measurement of fat content after rigor, due to the influence of membrane state on impedance, relating the good results of Slanger and Marchello (1994) [32] to the absence of membrane or extracellular compartment modifications immediately after slaughter and to the fact that the measurements were made at a stable temperature. The present results were obtained with samples kept at 2 °C for 12 days after slaughter and they are in the range of the results presented by Slanger and Marchello (1994) [32] and Madsen et al. (1997) [33]. Another question was raised by Ponnampalam et al. (2015) [26]—having tested meat samples with a fat content of 4.9% ± 2.5% wet weight; they concluded that bioimpedance spectroscopy was not suitable to predict fat content of meat samples low in fat. Altmann and Pliquett (2006) [35], comparing their percentage of 62.5% for correct classifications in beef above a threshold value of 2% with that of 80.2% obtained by Madsen et al. (1999) [36], for correct classifications above a threshold value of 3.5%, had already concluded that, depending on the level of IMF within a breed, low IMF was often predicted as too high or vice versa. Working with meat samples with a much wider range of fat content, Marchello et al. (1999) [37] also showed a remarkable difference in the accuracy of their impedance models to predict IMF. In fact, depending on the level of IMF, their impedance models explained 80% and 84–95% of the variation observed, respectively in beef trim and ground beef with 4.2 to 48.5% fat, but only explained 36% and 60–86% of the variation observed, respectively in beef trim and ground beef with less than 35% fat. In the present study, the IMF content ranged between 1.65% and 16.53%, and the best model still explained 79.3% of the variation observed, showing an RPD value of 2.2, which qualifies as a very good prediction model. Considering only samples above threshold values of 2%, as Altmann and Pliquett (2006) [35], and 3.5%, as Madsen et al. (1999) [36], there was no significant change in the predictive value of the best model for the present data set (data not shown).

The high correlation now obtained between BIA parameters and total collagen contrasts with the results of Lepetit et al. (2002) [38], that showed no trends between post-rigor impedance and the rank of muscles on their collagen content, finding support in the observation of Swatland (1980) [39] that a sheet of connective tissue has a similar impedance to meat. The fact is that, in the present study, the multiple regression analysis resulted in the development of a significant prediction model that, with an RPD value of 1.4 qualifies as a model of low to moderate predictive value.

While Hopkins and Wang (2012) [40], showed no significant correlation between impedance and 5-day shear force, Byrne et al. (2000) [41] had already reported a moderate to high correlation between impedance measurements and WBSF (r = 0.68, *p* < 0.001), and the present study showed a correlation significantly higher. The best predictive impedance model only explained 60.5% of the variation observed in WBSF, but its RPD value of 1.6 means that it can still be used for assessment of WBSF.

Concerning sarcomere length, Byrne et al. (2000) [41] showed no significant correlations with impedance measurements, but the present study showed a high positive correlation between sarcomere length and BIA parameters. This resulted in a prediction model still useful for assessment of sarcomere length (RPD = 1.7).

The moderate positive correlation now observed between WBSF and cooking losses compare to the moderate to high correlations (0.67 ≤ r ≤ 0, 79; *p* < 0.001) observed by Silva et al. (1999) [27] and, similarly to WBSF, cooking losses also showed a moderate correlation with BIA parameters. This contrasts with the results of Byrne et al. (2000) [41], showing just a few significant and poor correlations of cooking losses with impedance measured at different times. The fact is that the best prediction model now developed, with RPD = 1.2, showed to have poor predictive value.

The low to moderate correlations of total pigments and trichromatic coordinates with BIA parameters are in line with the conclusion of Byrne et al. (2000) [41] that the few significant correlations found with impedance measurements at specific times post mortem were of little value. This was confirmed by multiple regression analysis, showing RPD values between 1.1 and 1.4 for the best prediction models.

Callow (1936) [42] noticed that electrical impedance was related to ultimate pH and Bendall and Swatland (1988) [43] stated that electrical impedance was directly proportional to pH, with a curvilinear relationship, but Swatland (1995) [44] proposed that the relationship between impedance and pH was just indirect, due to a common relationship with ATP. While most studies in this area have focused on early detection of dark, firm and dry (DFD) meat and pale, soft and exudative (PSE) meat, Damez and Clerjon (2008) [45], besides pointing out that Guerrero et al. (2004) [46], for instance, showed no ability of electrical measurements in early detection of DFD, relate difficulties in the detection of PSE meats, during the period when rigor sets in, due to the fact that pH and temperature change rapidly in that period, while the associated structural changes that affect the electric properties of meat evolve more slowly. Concurring to the results of the present study, Byrne et al. (2000) [41], studying the correlations of pH measurements taken at 2, 5, and 7 h, with impedance measurements made between 7 h and 14 days post mortem, also obtained significant but low correlations between pH and impedance measurements. The fact is that the best impedance model now obtained must be qualified as a poor predictor of pH48 (RPD = 1.2).

## 5. Conclusions

The present results confirmed the potential interest of using BIA parameters for objective measurement of meat characteristics, since all traits of meat quality analyzed were correlated with BIA parameters, concurring to the type of correlation observed between each pair of traits. Moreover, the correlations with BIA parameters were validated with the development of significant BIA prediction models for all traits. From the present data set, it can be concluded that BIA was able to predict the IMF in LM samples. Concerning the other physicochemical traits of LM samples, it was also possible to develop models that provided useful information for the assessment of total collagen, sarcomere length and WBSF, but further research is needed to develop BIA equipment for use in meat processing plants.

## Figures and Tables

**Table 1:** Descriptive statistics for live weight (LW), dressing, hot carcass weight (HCW), longissimus thoracis et lumborum muscle (LM) physicochemical traits, and bioelectrical impedance (BIA) parameters (n = 52).

Trait	Mean	SD	Minimum	Maximum	CV (%)
LW (kg)	355.5	35.0	291.0	433.0	9.8
Dressing (%)	60	1.32	56	62	2.2
HCW (kg)	210.3	18.2	176.7	249.9	8.7
LM physicochemical traits					
IMF (%)	7.48	3.54	1.65	16.53	47.3
Total collagen (mg/g)	7.52	1.19	4.74	9.43	15.8
Cooking losses (%)	18.0	2.59	13.2	23.4	14.4
Sarcomere length (µm)	1.83	0.21	1.48	2.23	11.4
WBSF (kg/cm^2^)	5.51	0.99	3.72	7.62	18.0.
Total pigments (mg/g)	3.01	0.82	1.57	4.40	27.2
L*	31.0	3.89	25.1	41.8	12.5
a*	13.27	2.63	8.23	16.87	19.8
b*	8.99	1.47	3.49	12.1	16.3
pH48	5.95	0.24	5.55	6.61	4.0
BIA parameters					
Rs (Ω)	86.8	21.2	58.6	138.1	24.5
Xc (Ω)	108.9	34.1	65.1	161.5	31.4
Z (Ω)	139.4	39.5	89.7	212.1	28.4

SD = standard deviation; IMF = intramuscular fat; WBSF = Warner–Bratzler shear force; L*, a* and b* = trichromatic coordinates (brightness, red and yellow, respectively); pH48 = pH 48 h after slaughter; Rs = resistance; Xc = reactance; Z = impedance, calculated as (Rs^2^ + Xc^2^)^0.5^.

**Table 2:** Coefficients of correlation between the different longissimus thoracis et lumborum muscle (LM) physicochemical traits (n = 52) ^1^.

	Total Collagen (mg/g)	Total Pigments (mg/g)	Cooking Losses (%)	Sarcomere Length (µm)	WBSF (kg/cm^2^)	L*	a*	b*	pH48
IMF (%)	−0.869	0.386	−0.707	0.830	−0.798	0.642	0.424	0.708	−0.540
Total collagen (mg/g)		−0.252	0.647	−0.740	0.697	−0.565	−0.367	−0.654	0.476
Total pigments (mg/g)			−0.212	0.193	−0.417	0.223	−0.047	0.315	−0.334
Cooking losses (%)				−0.556	0.569	−0.432	−0.468	−0.604	0.414
Sarcomere length (µm)					−0.569	0.487	0.338	0.603	−0.362
WBSF(kg/cm^2^)						−0.457	−0.439	−0.700	0.640
L*							0.230	0.460	−0.319
a*								0.282	−0.280
b*									−0.471

IMF = intramuscular fat; WBSF = Warner–Bratzler shear force; L*, a* and b* = trichromatic coordinates (brightness, red and yellow, respectively); pH48 = pH 48 h after slaughter. ^1^ *p* < 0.01.

**Table 3:** Coefficients of correlation of bioelectrical impedance (BIA) parameters *with* longissimus thoracis et lumborum muscle (LM) physicochemical traits (n = 52) ^1^.

	Rs (Ω)	Xc (Ω)	Z (Ω)
IMF (%)	0.854	0.835	0.851
Total collagen (mg/g)	−0.709	−0.705	−0.716
Cooking losses (%)	−0.605	−0.598	−0.607
Sarcomere length (µm)	0.748	0.803	0.795
WBSF (kg/cm^2^)	−0.778	−0.747	−0.763
Total pigments (mg/g)	0.388	0.361	0.374
L*	0.541	0.567	0.565
a*	0.421	0.426	0.431
b*	0.610	0.569	0.590
pH48	−0.460	−0.489	−0.486

Rs = resistance; Xc = reactance; Z = impedance, calculated as (Rs^2^ + Xc^2^)^0.5^; IMF = intramuscular fat; WBSF = Warner–Bratzler shear force; L*, a* and b* = trichromatic coordinates (brightness, red and yellow, respectively); pH48 = pH 48 h after slaughter.^1^ *p* < 0.01.

**Table 4:** Best multiple regression models for predicting longissimus thoracis et lumborum muscle (LM) physicochemical traits (n = 52) ^1^.

TRAIT	Intercept	Rs	Xc	Z	k-Fold-*R*^2^	RMSE	RPD
IMF (%)	−5.117	0.129	0.014		0.793	1.631	2.2
Total collagen (mg/g)	10.529			−0.0215	0.513	0.839	1.4
Cooking losses (%)	24.041		0.041	−0.075	0.346	2.096	1.2
Sarcomere length (µm)	1.291		0.0049		0.644	0.127	1.7
WBSF (kg/cm^2^)	8.612	−0.0358			0.605	0.631	1.6
Total pigments (mg/g)	1.709	0.015			0.134	0.762	1.1
L*	23.958		0.064		0.308	3.232	1.2
a*	8.528	−3.149	−4.015	5.129	0.223	2.318	1.1
b*	5.338	0.042			0.360	1.174	1.3
pH48	6.382	0.300	0.377	−0.484	0.287	0.202	1.2

Rs = resistance; Xc = reactance; Z = impedance, calculated as (Rs^2^ + Xc^2^)^0.5^; IMF = intramuscular fat; WBSF = Warner–Bratzler shear force; L*, a* and b* = trichromatic coordinates (brightness, red and yellow, respectively); pH48 = pH 48 hours after slaughter. ^1^ All models are significant at *p* < 0.01.
